# Nitrilases in nitrile biocatalysis: recent progress and forthcoming research

**DOI:** 10.1186/1475-2859-11-142

**Published:** 2012-10-30

**Authors:** Jin-Song Gong, Zhen-Ming Lu, Heng Li, Jin-Song Shi, Zhe-Min Zhou, Zheng-Hong Xu

**Affiliations:** 1 The Key Laboratory of Industrial Biotechnology, Ministry of Education, Jiangnan University, Wuxi, People’s Republic of China; 2 Laboratory of Pharmaceutical Engineering, School of Medicine and Pharmaceutics, Jiangnan University, Wuxi, 214122, People’s Republic of China; 3 Laboratory of Bioactive Products Processing Engineering, School of Medicine and Pharmaceutics, Jiangnan University, Wuxi, 214122, People’s Republic of China

**Keywords:** Biocatalysis, Bioremediation, Carboxylic acid, Gene expression, Immobilization, Nitrilase, Nitrile, Purification, Strain screening, Surface modification

## Abstract

Over the past decades, nitrilases have drawn considerable attention because of their application in nitrile degradation as prominent biocatalysts. Nitrilases are derived from bacteria, filamentous fungi, yeasts, and plants. In-depth investigations on their natural sources function mechanisms, enzyme structure, screening pathways, and biocatalytic properties have been conducted. Moreover, the immobilization, purification, gene cloning and modifications of nitrilase have been dwelt upon. Some nitrilases are used commercially as biofactories for carboxylic acids production, waste treatment, and surface modification. This critical review summarizes the current status of nitrilase research, and discusses a number of challenges and significant attempts in its further development. Nitrilase is a significant and promising biocatalyst for catalytic applications.

## Background

### Green chemistry

Chemistry, as one of the oldest disciplines, has achieved great advances over the past centuries, owing to the discoveries by scholars in industry and academia worldwide [1]. In addition, chemistry has outstanding contributions to the development of the global economy, and the progress of human civilization. However, along with the diminishing fossil fuel reserves, and the increasing environmental pollution, research gradually focused on the development of economical, energy-saving, and environmentally benign chemical processes [1,2]. The global needs for clean production technologies, renewable raw materials and energy, and the treatment of hazardous chemicals and wastes presented new research challenges to both chemistry and biotechnology [3]. Hence, “green chemistry”, traced from the concepts of atom economy and E factors (E = wastes produced/product obtained), was introduced and became a strategic focus in both industrial and academic areas in the 1990s [2,4,5].

### Solution for green chemistry

Biocatalysis, the core of industrial biotechnology, is a solution to green chemistry because of its excellent catalytic efficiency and environmentally benign characteristics [1,3,6]. Its rapid development came from the recent advances in large-scale genome sequencing, directed evolution, protein expression, metabolic engineering, high throughput screening, and structural biology [7]. Biocatalysis has emerged as a promising tool for the catalytic processes using whole microbial cells, cell extracts, purified enzymes, immobilized cells, or immobilized enzymes as the catalysts for chemical synthesis [8,9]. Enzymatic transformations have been carried out for the production of a wide range of chemicals, including food ingredients, active pharmaceuticals, pharmaceutical and agrochemical intermediates, detergents, and other bulk chemicals [5,8,10,11]. Furthermore, instead of the conventional chemical synthesis, the biocatalytic routes are developed to meet the green chemistry requirements on cost-efficiency, waste reduction, energy consumption, and other synthesizing objectives (ideally, to synthesize target compounds with 100% yield and 100% selectivity) [1,5,12].

### Nitrile biocatalysis

Nitrile compounds, as nitrile biocatalysis reaction substrates, are widespread in the natural environment, and occur as cyanoglycosides, cyanolipids, ricinine, and phenylacetonitrile which were produced by plants [13]. In addition, nitriles are simple to synthesize using several pathways, such as the addition of a cyanide ion to alkyl halides, the Strecker reaction, the Sandmeyer reaction, and the reaction of aryl halides with copper cyanide [14]. Although most nitriles are highly toxic, mutagenic, and carcinogenic due to their cyano group, enzymatic hydrolysis of these compounds is a recognized method to avail a broad spectrum of useful amides, carboxylic acids, and so on [13,15]. Meanwhile, bioremediation with nitrile-converting enzymes is an efficient method for degrading highly toxic nitriles in environmental wastes and contaminants [16,17]. Nitrile catabolism comprises two distinct pathways [18]: (1) nitrilases (EC 3.5.5.1) directly convert nitriles to corresponding carboxylic acids and NH_3_; and (2) nitrile hydratases (NHases; EC 4.2.1.84) catalyze the formation of corresponding amides from nitriles, and amidases (EC 3.5.1.4) subsequently hydrolyze amides to carboxylic acids and NH_3_. This review focuses primarily on nitrilase research.

### Nitrilase

**Figure 1:**
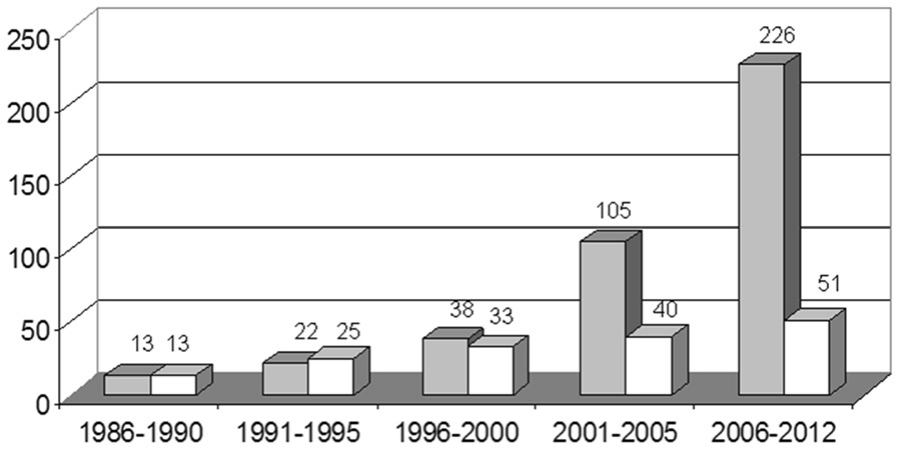
**Number of publications on nitrilase research.** Articles (■) were assessed according to Web of Science; patents (□) according to Espacenet search conducted at the end of July 2012.

In recent years, nitrilase-mediated biocatalysis has attracted substantial attention from scholars and entrepreneurs. Several studies on nitrilase application in chemical synthesis were carried out, since the first nitrilase was discovered in the early 1960s [19]. Over the past five decades, various nitrilase-producing organisms, including bacteria, filamentous fungi, yeasts, and plants were described [13,20-22], and some of these microbial cell factories were utilized for the commercial production of carboxylic acids in industrial scale. The success of nicotinic acid (Lonza, China) and (*R*)-(−)-mandelic acid (Mitsubishi Rayon, Japan; BASF, Germany) industrial production using nitrilase proved the great economic potential of nitrilase [9,23-25]. Intensive research on nitrilase-mediated biocatalysis has occurred in the past decades, as shown by the increasing publications on enzymatic nitrile hydrolysis catalyzed by nitrilase (Figure 1). The present study summarizes the current scientific knowledge on nitrilase and thus lays the foundation for prospective industrial nitrilase applications. This review will concentrate on nitrilase occurrence, catalytic reaction mechanism, reaction properties, enzyme purification, immobilization, nitrilase gene cloning, molecular modification, and its industrial applications. Furthermore, this review will discuss the challenges faced by researchers and the future prospect in this field.

### Nitrilase sources

Nitrilase activities exist extensively in nature, especially in bacteria, filamentous fungi and plants, although it is difficult to assess the actual distribution frequency of these organisms. On the other hand, the microbial nitrilase as a “green catalyst” for production of useful compounds with commercial value and other catalytic applications is easier to control from viewpoint of biotechnological application. Based on the substrate specificity of nitrilases from different sources, they were classified into three major categories, namely aromatic nitrilase, aliphatic nitrilase and arylacetonitrilase [26].

#### Bacteria

Since the 1960s, studies on nitrilase focused mainly on the bacteria that produce this enzyme. The first bacterial strain to hydrolyze the ricinine nitrile group was isolated from the soil, and was identified as belonging to the *Pseudomonas* genus based on primary morphological characteristics [27]. Ricinine nitrilase from this *Pseudomonas* strain was then purified, and characterized [28]. Henceforth, several bacteria from the genera of *Rhodococcus*, *Nocardia*, *Acinetobacter*, *Alcaligenes*, *Pseudomonas*, *Corynebacterium*, and so on, were reported to display nitrilase activity (Table 1); among which, several nitrilases were purified, characterized, immobilized, gene cloned, over-expressed in host strains, and applied in industrial processes. The success of nitrile-converting enzymes from *R. rhodochrous* J1, which were utilized in industrial production of amides and carboxylic acids substantially boosted the confidence of researchers and entrepreneurs in this area. Thus, during the last two decades, the number of reported bacteria with nitrilases rapidly increased yearly.

**Table 1:** The reported bacteria with nitrilase activity in previous literature

**Bacteria**	**Formation type**	**Molecular weight (kDa)**	**Optimum pH / temperature (°C)**	**Stability pH / temperature (°C)**	**Substrate specificity**	***K*m (M)**	**Reference**
*Pseudomonas* sp.	Inducible	-	-	7.4-8.8 / -	N-Methyl/ethyl-3-cyano-4- methoxy-2-pyridone	1.9×10^-5^	[27]
*Nocardia* sp. NCIB 11216	Inducible	560	8 / -	-	Aromatic nitrile	-	[29]
*Brevibacterium* strain R312	Constitutive	-	7 / 35	- / below 30	-	2.5×10^-2^	[30]
*Pseudomonas* sp. 13	Inducible	1,000	9 / 55	7-11 / below 60	β-Cyano-L-alanine	8.3×10^-2^	[31]
*Acinetobaeter* sp. APN	Inducible	-	-	-	α-Aminonitriles	3.4×10^-2^	[32]
*R. rhodochrous* J1	Inducible	78	7.6 / 45	- / 20-50	Aliphatic, aromatic nitriles	-	[33,34]
*Comamonas testosteroni* sp.	Inducible	-	-	-	Aliphatic nitriles	-	[35]
*A. faecalis* JM3	Inducible	275	7.5 / 45	7-8 / 20-50	Arylacetonitriles	3.3×10^-4^	[36]
*Acinetobacter* sp. AK226	Inducible	580	8 / 50	5.8-8 / below 60	Aliphatic, heterocyclic nitriles	1.2×10^-2^	[37]
*A. faecalis* ATCC 8750	Inducible	460	7.5 / 40-45	6.5-8 / below 50	Arylacetonitriles	5.8×10^-3^	[38]
*R. rhodochrous* PA-34	Inducible	45	7.5 / 35	- / below 35	Aromatic, aliphatic nitriles	4.5×10^-2^	[39]
*R. rhodochrous* K22	Inducible	650	5.5 / 50	- / below 55	Aliphatic nitriles	1.9×10^-2^	[40]
*Klebsiella ozaenae*	Constitutive	37	9.2 / 35	-	Bromoxynil	3.1×10^-4^	[41]
*Bacillus pallidus* Dac521	Inducible	600	7.6 / 65	6-9 / below 65	Aromatic nitriles	9.2×10^-4^	[42]
*R. rhodochrous* NCIMB 11216	Inducible	45.8	8 / 30	-	Aromatic nitriles	2.1×10^-2^	[43]
*P. fluorescens* DSM 7155	Inducible	130	9 / 55	-	Arylacetonitriles	8.7×10^-5^	[44]
*N. globerula* NHB-2	Inducible	-	-	-	Aromatic, unsaturated aliphatic nitriles	-	[45]
*P. putida*	Inducible	412	7 / 40	6.5-8 / below 50	Arylacetonitriles	1.3×10^-2^	[46]
*Pyrococcus abyssi* GE5	Inducible	60	7.4 / 80	4.5–8.5 / 60-90	Aliphatic dinitriles	9.5×10^-3^	[47]
*Acidovorax facilis* 72W	Inducible	570	- / 65	5-10 / below 60	Aliphatic dinitriles	5.6×10^-2^	[48]
*Rhodococcus* sp. NDB1165	Inducible	-	8 / 45	- / below 50	Aromatic and unsaturated aliphatic nitriles	0.1	[49]
*Bradyrhizobium japonicum* USDA110	Inducible	455	-	-	Mandelonitrile, phenylacetonitrile	2.6×10^-4^	[50]
*P. aeruginosa* 10145	Inducible	-	-	-	Aromatic nitriles	-	[51]
*Halomonas nitrilicus* sp. nov.	Inducible	-	-	-	Arylaliphatic nitriles	-	[52]
Bacillus subtilis ZJB-063	Constitutive	-	-	-	Arylacetonitriles	-	[53]
*Alcaligenes* sp. ECU0401	Constitutive	376	8 / 40	- / below 50	Aliphatic and aromatic nitriles	2.2×10^-2^	[54]
*P. fluorescens* Pf-5	-	138	7 / 45	- / below 65	Dinitriles	1.8×10^-2^	[55]
*Streptomyces* sp. MTCC 7546	Inducible	-	-	-	Aliphatic nitriles	-	[56]
*Arthrobacter nitroguajacolicus* ZJUTB06-99	Inducible	-	6.5 / 40	- / below 50	Aliphatic and aromatic nitriles	1.2×10^-2^	[57]
*R. erythropolis* ZJB-0910	Inducible	-	7.5 / 30	- / below 30	β-Hydroxy aliphatic nitrile	1.0×10^-2^	[58]
*Geobacillus pallidus* RAPc8	Inducible	600	-	-	Aromatic nitriles	-	[59]
*A. faecalis* ZJUTB10	Inducible	-	7.7–8.5 / 35	- / below 35	-	-	[60]
*A. faecalis* MTCC 10757	Inducible	-	8 / 35	-	Aliphatic and aromatic nitriles	-	[61]

Note: “-” indicates no data available.

#### Filamentous fungi

In earlier studies, nitrilase activity was reported in several fungal strains from the genera of *Fusarium*, *Gibberella*, *Aspergillus*, and *Penicillium*, which can hydrolyze indole-3-acetonitrile (IAN) into indole-3-acetic acid (IAA) [19]; however, little information is available on fungal nitrilase in the following decades. More precisely, only a *F. solani* was reported capable of degradation of the herbicides 3,5-dibromo-4-hydroxybenzonitrile (bromoxynil) and 3,5-diiodo-4-hydroxybenzonitrile (ioxynil) by Hsu and Camper [62]. Thereafter, nitrilase from *F. solani* IMI 196840 [63], isolated from bromoxynil-treated soil and can grow with benzonitrile as its sole carbon and nitrogen source, was purified and characterized. The significance of nitrilase from *F. solani* IMI 196840 in the breakdown of herbicides in the environment was discussed. Since then, *F. solani* nitrilase was the only uniquely characterized fungal nitrilase for more than ten years until the appearance of *F. oxysporum* f. sp. *melonis* nitrilase in 1989 [64]. Later, a group from Czech Republic tackled nitrilase research, especially fungal nitrilases. Nitrilases from *A. niger* K10 and *F. solani* O1, used by this group, proved to be promising biocatalysts in nitrile transformation [65]. Both these new fungal nitrilases were purified, immobilized, and used to synthesize carboxylic acids [66-68]. Moreover, the aforementioned *F. solani* IMI 196840 nitrilase was further investigated [69]. Table 2 shows some reported filamentous fungi with nitrilase activity.

**Table 2:** The reported filamentous fungi with nitrilase activity in the previous literature

**Filamentous fungi**	**Formation type**	**Molecular weight (kDa)**	**Optimum pH / temperature (°C)**	**Stability pH / temperature (°C)**	**Substrate specificity**	***Km*(M)**	**Reference**
*A. furmigatus*	Inducible	-	-	-	α-Aminophenylacetonitrile	-	[70]
*F. oxysporum* f sp. *melonis*	Inducible	550	- / 40	6-11 / below 40	Aromatic nitriles	1.7×10^-2^	[64]
*A. niger* K10	Inducible	>650	8 / 45	7.2-9 / below 30	Aromatic nitriles	4.7×10^-3^	[66]
*F. solani* O1	Inducible	580	8 / 50	7 -9 / 35-50	Aromatic nitriles	1.4×10^-3^	[67]
*F. solani* IMI 196840	Inducible	550	8 / 45	7.8-9.1 / below 50	Aromatic nitriles	-	[63,69]

Note: “-” indicates no data available.

#### Yeasts

More than 60 nitrile-metabolizing yeasts, including the species of *Candida*, *Pichia*, *Saccharomyces*, *Hanseniaspora*, *Debaryomyces*, *Geotrichum*, *Williopsis*, *Torulopsis*, *Exophiala*, *Kluyveromyces*, *Aureobasidium*, *Cryptococcus*, and *Rhodotorula*, were isolated from cyanide treatment bioreactor, fermented foods, and the soil [71,72]. Nonetheless, most of these species contained the nitrile hydratase-amidase system, rather than the nitrilase system, for nitrile biocatalysis. Only few yeasts display nitrilase activities.

Fukuda et al. used an enrichment medium with 0.1% D,L-α-hydroxy isovaleronitrile as the sole nitrogen source and obtained a *T. candida* GN405 strain that can effectively synthesize the optically active α-hydroxyacids from D,L-α-hydroxynitrile compounds, but failed to hydrolyze D,L-α-aminonitriles [73]. Yeast nitrilase-mediated biotransformation tends to occur under acidic conditions, out of consideration for the optimal pH for the growth of yeast strains (generally, 4.0-7.0). Thus, yeast nitrilase was suitable for the hydrolysis of hydroxynitriles and aminonitriles, which spontaneously decomposed under neutral conditions, but were more stable at acidic pH. The nitrile-hydrolyzing yeast *E. oligosperma* R1, isolated in a selective medium of pH 4.0, has significant stability and excellent catalytic efficiency for hydroxynitriles under acid conditions [21,74]. Rezende’s study indicated that the nitrilase system of *Cryptococcus* sp. UFMG-Y28 could be induced in the presence of benzonitrile as the sole nitrogen source. Moreover, this strain was also found to produce nitrile hydratase (NHase) and amidase when grown on other nitriles as nitrogen source [75].

#### Plants

Plant nitrilase is an important enzyme in the nitrilase superfamily. Studies on plant nitrilases in its early days mainly focused on their potential in converting IAN into IAA, which is important in promoting elongation growth of plants. However, the function of nitrilases in plant auxin biosynthesis is still unclear [22]. The first plant nitrilase that hydrolyzed IAN to IAA in barley, was described by Thimann in 1964 [19]. Furthermore, several plant species were reported to contain various nitrile compounds [71], indicating that nitrilases widely exist in plants.

Nitrilase from Chinese cabbage seedlings was partial purified, and its significance for IAA synthesis was discussed [76]. The *Brassica rapa* nitrilases showed excellent activities for various aliphatic and aromatic nitriles, but poor for IAN [77]. Arylacetonitriles, such as 4-hydroxy and 4-methoxy phenylacetonitrile, were proven good substrates for *Sinapis alba* and *S. arvensis* nitrilase (the latter also showed NHase activity) [78]. *Arabidopsis thaliana*, a member of the *Brassicaceae* family, expressed four kinds of nitrilases (AtNIT1 to AtNIT4). AtNIT1 to AtNIT3, which can convert IAN into IAA, are very similar to each other, but less similar to AtNIT4 [79,80]. AtNIT4 has only 65% amino acids identical to that of the AtNIT1 to AtNIT3. The AtNIT4 gene is located on chromosome 5, whereas the AtNIT1 to AtNIT3 genes are clustered on chromosome 3, and have more than 80% sequence identities [81]. Moreover, NIT4 gene is found to exist in all plants (from mosses on) [22,80]. NIT4 enzymes are mostly very specific for β-cyano-L-alanine, which is an intermediate product in the plant cyanide detoxification pathway [79,82].

Recent studies on plant nitrilases demonstrated their cyanide detoxification function. Cyanide, which is highly toxic, is produced as by-product in biosynthesis of the plant hormone ethylene in all plants [83]. On the other hand, the other possible sources of cyanide in some plants was cyanogenic glycosides, which are plant defense compounds and can be degraded into an aldehyde, ketone or hydrogen cyanide by β-glucosidases and α-hydroxynitrilases [22]. Therefore, cyanide detoxification is necessary for plant growth. *Lotus japonicus* contains two cyanogenic α-hydroxynitrile glycosides. Piotrowski et al. successfully cloned a nitrilase gene from *L. japonicus*, which displayed remarkable β-cyanoalanine hydrolyzing activity [22]. The nitrilase heterodimers were probably involved in the cyanogenic glucoside catabolism of sweet almonds and sorghum [84]. Furthermore, several additional plant nitrilases have evolved, which might be involved in the catabolism of cyanogenic glycosides [83] or in the catabolism of glucosinolates [78,80,81].

### Nitrilase obtaining manners

In all these cases of biocatalytic applications, improving the catalytic properties of existing enzymes or finding the potential of new ones is strongly demanded. Considering the new ones, they should first be obtained from the natural environment before an in-depth study and practical application. Thus, various pathways to acquire the target nitrilase were introduced. According to Yamada, one of the leading founders in the area of industrial biocatalysis, searching for better biocatalysts to improve productivity is still of great significance even when the industrial process functions well [85]. Hence, the importance of continuing the screening for microorganisms of interest always remains.

#### Conventional screening

Traditionally, nitrilase-producing microorganisms were screened in a selective medium with nitriles as the sole source of nitrogen or/and carbon, where only the strains harboring nitrile-degrading activity can grow. For example, Shen et al. isolated a novel *A. nitroguajacolicus* from soil samples with acrylonitrile as the sole nitrogen source, which can convert acrylonitrile to acrylic acid [57]. An enrichment medium, with glucose as the carbon source and phenylacetonitrile as the sole source of nitrogen, was applied to select the acidotolerant nitrilase-producing yeasts. A black yeast was isolated and identified as an *E. oligosperma* strain [21]. A fungal *F. solani* strain was isolated by Harper from the bromoxynil-treated soil with benzonitrile as sole carbon and nitrogen source and is a promising degrader of nitrilic herbicides [63].

Nitrilase activity assay is based on NH_3_ analysis, which is equimolarly produced with acids from the bioconversion reaction. NH_3_ determination is usually performed using the phenol-hypochlorite reaction [86], as well as the CoCl_2_-based [87], Nessler [88], and Indophenol methods [89]. Mass spectrometry, nuclear magnetic resonance (NMR) spectroscopy, liquid and gas chromatography are often used as the quantitation methods for substrate and product assays [90].

#### High throughput screening

Conventional screening is an effective method for obtaining nitrilase-producing microorganisms; however, it is a time-consuming and tedious work. Furthermore, quantitative detection methods are expensive and slow [90]. Consequently, the high-throughput screening method was developed and has gained increasing popularity in the search for potential biocatalysts in recent years. The assays are based on fluorogenic and chromogenic substrates, or pH indicator methods. Santoshkumar et al. recently developed a pH indicator method to rapidly screen aliphatic nitrile-degrading bacteria, and found that some indicators, such as phenol red, bromothymol blue, and phenolphthalein, were sensitive to the liberation of ammonia from the nitrile-utilizing bacteria [91]. He et al. first proposed a simple and accurate high-throughput screening method based on ferric hydroxamate spectrophotometry. They showed that the accuracy of this method for determining carboxylic acid was extremely high compared with the HPLC-based method [92]. A novel time-resolved luminescent probe of *o*-hydroxybenzonitrile derivatives was utilized in high-throughput detection of nitrilase activity [93]. Higher sensitivity with this probe was observed because the time-resolved property of the luminescence can reduce the background from other proteins. A rapid and sensitive fluorometric method was proposed by Banerjee et al., which employed *o*-phthaldialdehyde-2-mercaptoethanol with nitrilase reaction solution to form a fluorochrome [94], and the results further supported the established Berthelot method. Therefore, other simple, sensitive, and/or accurate assays should be developed to display the importance of high-throughput screening.

### Structure and catalytic properties of nitrilase

The superficial understanding on the protein structure and catalytic properties of nitrilase is the main barrier for molecular manipulation, such as rational design. Therefore, the determination of the exact protein structure, and the catalytic mechanism of nitrilase comes into prominence, with the rising requirement for molecular operations.

**Figure 2:**
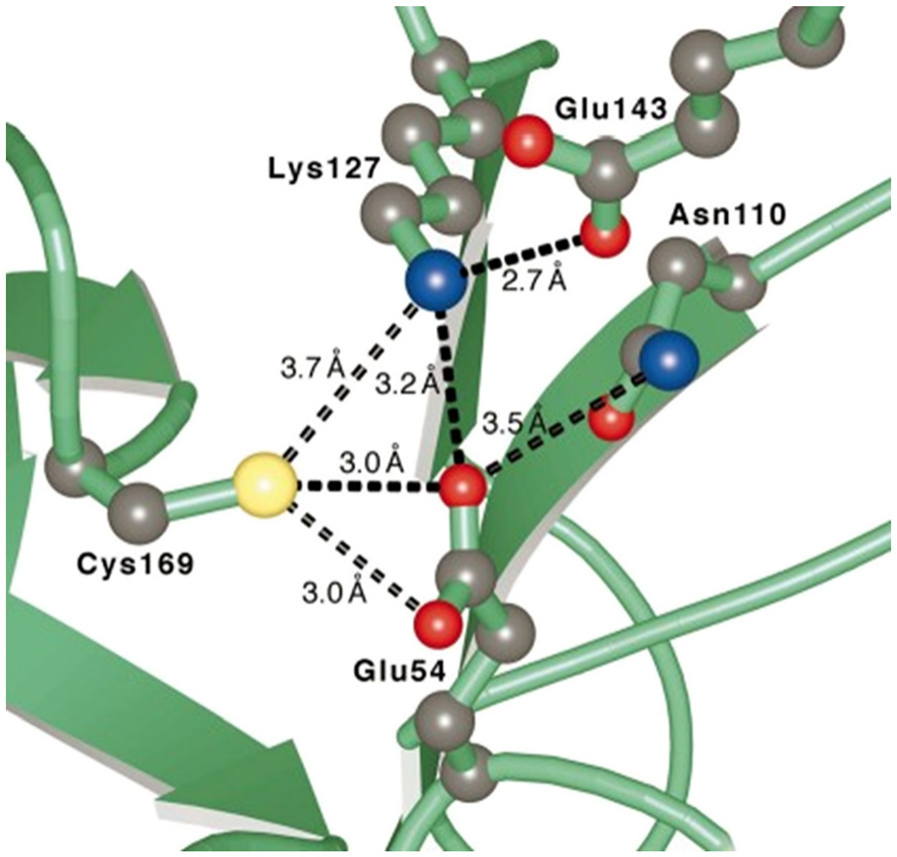
**Putative nitrilase active site **[95]. The related residues of Glu54, Lys127 and Cys169 were considered functionally important as a catalytic triad in the nitrilase superfamily.

According to Brenner and his colleagues, the structure of the nitrilase protein is a novel α-β-β-α sandwich fold, with a triad of residues, Glu-Lys-Cys (Figure 2), which is essential in the function of its active site and enhances the performance of nitrilase [95]. The results achieved by Sewell’s group confirmed that the nitrilase from *R. rhodocrous* J1 have three homo-oligomeric structural forms: a dimer, a 480 kDa complex, and an extended helix [96]. The J1 nitrilase model was generated, and this enzyme has an extended C-terminus and two significant insertions that intersperse in the spiral oligomer and lead to spiral elongation. Meanwhile, the 3D structures of these helical homooligomers were determined by electron microscopy. The 3D stain envelope was consistent with cyanide dihydratase from *P. stutzeri*, a member of the nitrilase superfamily [96,97]. Nitrilase from *G. pallidus* RAPc8 showed various structural forms, such as crescent-like, c-shaped, circular, and “figure-8” shapes through electron microscopy and image classification [59]. Far-UV circular dichroism (CD) spectroscopy was employed to determine the secondary protein structure of nitrilases from *F. solani* O1, and *F. solani* IMI 196840 [69]. Both nitrilases harbored almost identical contents in their secondary structure, consisting of 30% α-helix, 21% β-sheet, 16% turn, and 33% other structures.

**Figure 3:**
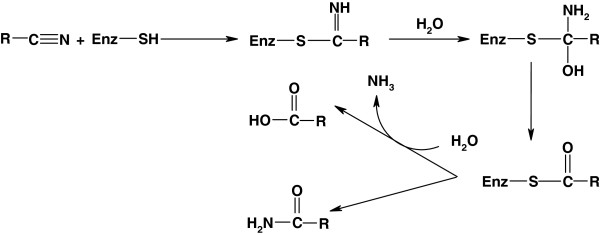
Proposed mechanism for nitrilase-mediated biocatalysis.

The catalytic properties of the nitrilase transformation reaction are widely investigated by several groups. In the early 1960s when the first nitrilase was found by Mahadevan et al. [19], it has postulated that the nitrile carbon bearing a fractional positive charge was subjected to a nucleophilic attack, probably by one of the two SH groups on the nitrilase. The resulting imine was then hydrolyzed to corresponding ketone while having NH_3_ as a by-product. Acyl-enzyme was then hydrolyzed by the addition of H_2_O, and finally liberated the carboxylic acid along with the regenerated enzyme. On the same year, Hook and Robinson demonstrated that a similar mechanism was also responsible for the ricinine hydrolysis through the ricinine nitrilase [28]. The proposed mechanism has been widely accepted by researchers to date (Figure 3) [13,98,99]. However, recent studies demonstrated that amide compounds might also be formed in the course of nitrile hydrolysis by nitrilase. Piotrowski et al. reported for the first time the NHase activity shown by the nitrilase from *Arabidopsis thaliana*, which can convert β-cyano-L-alanine into aspartic acid and asparagine (with >60% asparagine formation) [79]. Hence, the authors predicted that this reaction was contributed by a new type of NHase activity, different from the known mechanism of “classical” NHases. This NHase activity is probably attributed to the advanced release of an enzyme-bound substrate after the addition of the first H_2_O. Subsequently, amide was produced based on the belated delivery of the second H_2_O. Afterward, the significant amide formation under nitrilase control was studied in terms of steric requirements and electron density [100], which showed that the absolute α-carbon configuration in the substrate had a great effect on the acid/amide ratio. Furthermore, a low temperature and increased pH prompted the reaction towards amide. Meanwhile, the electron-withdrawing substituents at the α-position tended towards amide formation.

Some nitrilases possessed a subunit size between 30 kDa and 45 kDa, and must form heterocomplexes to gain catalytic activity. *Poaceae* nitrilase (NIT4 enzyme) activity was entirely derived from the formation of heteromeric complexes [83]. This was different from other plant nitrilases. The complexes were consisted of two NIT4 isoforms (NIT4A and NIT4B). Stevenson et al. found that the binding of an aromatic ring in the substrates can lead to the aggregation of the *Rhodococcus* ATCC 39484 nitrilase [101]. Such substrate-induction activated the enzyme by the formation of a 560-kDa complex through the association of the 47-kDa monomer. HPLC gel filtration can detect an association/dissociation equilibrium. The same aspect was also observed for the *R. rhodochrous* J1 nitrilase, where a number of salts and organic solvents can improve the association [102]. Enzyme forms were unstable during nitrilase assembly and nitrilase activities were only detected in the dimer and decamer.

### Factors influencing nitrilase production and its bioconversion

Several factors conspicuously influence the enzyme activity or biomass formation, including culture conditions, such as carbon source, nitrogen source, inducer and metal ion, and the bioconversion conditions, such as temperature, pH, enzyme modifier, and organic solvent.

### Factors that influence nitrilase production

#### Carbon and nitrogen source

Commonly, carbon and nitrogen sources have remarkable effects on enzyme yield. These sources are significant in cell proliferation and cellular enzyme biosynthesis [103]. Furthermore, in some situations, the nature and concentration of these nutrients regulated enzyme formation through physiological events, such as the catabolic repression from glycerol, glucose, and fructose. A foremost example was *P. putida* MTCC 5110, in which little nitrilase production was obtained when glycerol was utilized as the carbon source, although it supported an excellent biomass [104]. Sodium acetate, starch, glucose, and mannitol are the optimum carbon sources, whereas peptone, yeast extract, and casamino acid are the optimum nitrogen sources for nitrilase formation in *Alcaligenes* sp., *A. faecalis*, *A. nitroguajacolicus*, *P. putida*, *Rhodococcus* sp., *E. oligosperma*, as reported previously [61,105-107].

#### Inducer

To the best of our knowledge, most nitrilases are inducible by substrates, products, and their analogs, as shown in Tables 1 and 2. However, little is known about constitutive nitrilases, aside from those of *Brevibacterium* R312, *K. ozaenae*, *B. subtilis* ZJB-063, and *Alcaligenes* sp. ECU0401 as reported previously [30,53,105,108]. In addition, the multi-enzyme system including nitrilase and NHase/amidase in *Amycolatopsis* sp. IITR215 is constitutive [109]. In general, certain nitriles, amides, and carboxylic acids function as nitrilase inducers. ε-Caprolactam, first found as a powerful inducer of the *R. rhodochrous* J1 nitrilase, also results in the effective induction in some other *Rhodococcus* strains [110]. Nevertheless, *Rhodococcus* sp. NDB 1165 nitrilase was induced by propionitrile [49]. Benzonitrile was used as the inducer of nitrilase from *P. aeruginosa* 10145, in which cell growth was inhibited by the addition of benzonitrile at the beginning and thus the inducer was added after cultivation for 24 h [51]. The effects of different inducers on substrate specificity of *A. faecalis* MTCC 10757 were investigated by Nageshwar et al., and acrylonitrile was the most preferred inducer and substrate [61]. With regard to fungal nitrilase, 2-cyanopyridine was reputed a universal inducer in filamentous fungi, such as those from the genera of *Aspergillus*, *Fusarium*, and *Penicillium*[20]. The total nitrilase activity of *F. solani* O1, in the presence of 2-cyanopyridine, can be enhanced by a hundred fold compared with other inducers [111]. However, our experiments demonstrated that caprolactam was also a useful inducer for fungal nitrilase from the *Fusarium* genus [86]. Detailed work on caprolactam induction was attempted to further improve the fungal nitrilase formation.

#### Metal ion

Metal ions, such as Fe^3+^ or Co^2+^, were essential for the formation of NHases [112]; however, nitrilases do not require any metal ions as co-factor for their activity. These nitrilases have the catalytic cysteine residues at or near their active site, as forementioned. As yet, few studies reported the effect of metal ions on nitrilase production. Co^2+^, Hg^2+^, and Ag^+^ may inhibit the specific activity of nitrilase, and Cu^2+^ can hinder cell growth. Such an occurrence can be found in *R. rhodochrous* J1 [113] and *P. putida* MTCC 5110 [104]. Nevertheless, a reverse occurrence was unusually observed in *Alcaligenes* sp. ECU0401, in which Cu^2+^ supported both a higher biomass and more nitrilase production [105]. However, the exact mechanism for the enhancement of nitrilase activity by metal ions is still unclear. Although most metal ions promoted neither biomass nor enzyme formation in the previous limited studies, investigation on the effects of metal ions is still needed.

### Factors that influence nitrilase bioconversion

#### Temperature and pH

Temperature and pH often affect the structure and the nature of an enzyme, thereby further influence enzyme activity. Enzymatic reactions are generally performed under relatively mild conditions. Tables 1 and 2 showed that the optimum pH and temperature for the bioconversion reaction by nitrilase from most bacteria and fungi are placed in the pH range of 7.0-8.0 and in the temperatures ranging from 30°C to 55°C, respectively. Few nitrilases can function under extreme conditions. An alkali resistant nitrilase from *Pseudomonas* sp. 13 was characterized [31] and proved to have the optimum pH of 9.5 in the presence of 10 mM 2-mercaptoethanol. Rustler et al. [74] demonstrated that *E. oligosperma* nitrilase can hydrolyze the α-hydroxynitriles under acidic conditions.

Temperature is one of the most crucial factors for enzymatic reaction rate. The enzymatic reaction rate increases with the increase in reaction temperature because the molecular free energy increases resulting from the rising temperature, which can make more efficient collisions between the molecules [57]. On the contrary, excessively higher temperature leads to protein denaturation and thus the loss of enzyme activity. Thermoactive nitrilase from the anaerobic archaeon *P. abyssi* that grows optimally at 100°C, is an exception [47].

#### Stability

Operational stability was proven as one of the key requirements for biocatalyst on a commercial scale. The stability of nitrilases has been widely investigated, including their thermostability and pH stability. The half-life is an important evaluation index of biocatalyst stability. Previous studies revealed that the nitrilases from *P. putida*[114], *Microbacterium paraoxydans*[114], *M. liquefaciens*[114], *A. nitroguajacolicus*[57], and *A. faecalis*[60] had half-lives of 27.3 h, 1.7 h, 5.4 h, 157.6 h, and 14 d at 30°C, respectively. Remarkably, a highly thermostable nitrilase, with a half-life of 25 h at 70°C, 9 h at 80°C, and 6 h at 90°C, was purified from *P. abyssi*[47]. The half-lives of fungal nitrilase from *F. solani* O1 were 178 h and 4.4 h at 28°C and 45°C, respectively [111]. Meanwhile, our studies observed the half-lives of 231.1 h and 72.9 h at 30°C and 40°C for *Gibberella* nitrilase, respectively (data not shown). Nonetheless, Goldlust et al. [64] revealed that heating for 10 min at more than 40°C would result in the inactiviation of nitrilase from *F. oxysporum* f sp. *melonis*.

Similarly, most nitrilases displayed low stability and rapidly became inactivated in extreme pH conditions. The stability of the nitrilase from *P. putida* MTCC 5110 was determined at different pH values ranging from 6.5 to 8.0 [46]. Nitrilase stability of this enzyme was maintained at pH 7.0 with a half-life of 110 min, but rapidly decreased as pH varied above and below pH 7.0, and exhibited the half-lives of 36, 87, and 53 min at pH 6.5, 7.5, and 8.0, respectively. However, recombinant nitrilase from *Alcaligenes* sp. ECU0401 showed excellent stability at pH 7.0 and 8.0, with half-lives of 382 and 43 h, respectively [54]. Therefore, such a robust biocatalyst may have great potential for its industrial application in nitrile biocatalysis.

A concentrated enzyme solution was adopted by Griengl et al. to synthesize cyanohydrins with hydroxynitrile lyase in two-phase systems to improve the enzyme stability [115]. On the other hand, immobilization, as discussed below, is favorable to biocatalyst stability probably because immobilized cells were protected against the toxic effects of the substrates [15].

#### Organic solvent

Organic solvents are a kind of co-solvent, which have been recognized to increase the solubility of substrates or products in the bioconversion reaction. The enzymatic reactions in organic solvents, such as the lipase-mediated reaction, were extensively reported. In recent years, the degree of resistance to organic solvents for nitrilase has also been frequently investigated because of the poor solubility of most nitriles. Heinemann et al. observed the presence of methanol increased the reaction rate of the recombinant nitrilase from *Synechocystis* sp. PCC6803 [116]. The maximal conversion of this nitrilase was achieved in the presence of 40% (v/v) hexadecane. Previous studies on *F. solani* O1 suggested that the nitrilase was fairly stable in selected organo-aqueous media [67]. More than half of its initial activity was maintained in the presence of 5%-50% of n-hexane or n-heptane, or 5%-15% of xylene or ethanol. Moreover, full activity of *Pseudomonas* sp. DSM 11387 nitrilase was retained in the presence of 50% (v/v) hexadecane [117]. In addition, the presence of large amounts of organic solvents in the oxynitrilase mediated transformation reaction was reported to suppress the reversible chemical reaction [118]. The organic solvent increased the tendency of the chemical equilibrium to synthesize more products.

#### Enzyme modifier

Numerous enzyme modifiers, including inhibitors, stabilizers and metal ions, obviously affect enzyme activity. Nitrilases have catalytically conserved cysteine residues that contain the thiol group. Thiol-binding reagents, such as Cu^2+^, Ag^+^, Hg^2+^, N-ethylmaleimide, 5, 5-dithio-bis (2-nitrobenzoic acid), iodoacetamide, iodoacetic acid, *p*-chloromercuribenzoate, and *p*-hydroxymercuribenzoate, were proven strong inhibitors of nitrilase activity in literature [39,46,47,53,69]. High sensitivity to these reagents indicates the importance of thiol group in the catalytic activity. Carbonyl reagents such as hydroxylamine, phenylhydrazine, and semicarbazide, were reported to have no significant inhibitory effect on nitrilases from *P. putida* MTCC 5110 [46] and *R. rhodochrous* PA-34 [39], but inhibited nitrilase activities of *R. rhodochrous* J1 [34] and *A. faecalis* JM3 [36]. Previous study showed that metal chelating agents, such as ethylene diamine tetraacetic acid (EDTA), sodium azide, 8-hydroxyquinoline, and *o*-phenanthroline, had little or no influence on nitrilase activity [46,47,55,57]. However, EDTA greatly activated the *Alcaligenes* sp. nitrilase activity at 1 mM, but had a slight inhibitory effect at 5 mM [54]. This phenomenon further confirmed that nitrilases do not require metal ions as co-factor. Moreover, the effect of reducing agents, including dithiothreitol, 2-mercaptoethanol, D-cysteine, L-cysteine, ascorbic acid, and reduced glutathione on enzyme activity was determined, and their positive impact on nitrilase activity was observed in *P. abyssi*[47]. However, these agents had no advantageous effects on the activity of nitrilases from *Pseudomonas* strains [46,55]. In addition, dithiothreitol caused the inhibition of nitrilase activity from filamentous fungi, such as *F. solani* O1 and *A. niger* K10 [66,67].

#### Substrate and product concentration

Nitrile compounds are highly toxic and harmful to the nitrilase protein, which makes the substrate concentration become one of the limiting factors for the biotransformation process. However, from another standpoint, a higher substrate concentration around the enzyme produces a higher reaction rate because of chemical equilibrium [119]. In addition, at least ≥50 g·L^-1^ product concentration and excellent gram product/gram catalyst yield are necessary for industrial scale [120]. Almatawah et al. determined the effects of substrate and product concentration on the bioconversion of 3-cyanopyridine into nicotinic acid by *B. pallidus* Dac521 nitrilase [121]. The results demonstrated that both 3-cyanopyridine and nicotinic acid inhibited the hydrolysis reaction at concentrations greater than 200 mM. (*RS*)-mandelonitrile is hypertoxic; however, no substrate inhibition of *A. faecalis* ZJUTB10 nitrilase was observed under the (*RS*)-mandelonitrile concentration from 10 mM to 50 mM [60]. Furthermore, the recombinant nitrilase of *Alcaligenes* sp. showed a high substrate tolerance and was capable of withstanding 200 mM (*RS*)-mandelonitrile as substrate [122].

### Separation and purification of nitrilase

Nitrilases are typically intracellular enzymes that are fragile and susceptible to outside environment. Therefore, purification of nitrilases from microbial cells is more difficult than extracellular enzymes. All purification procedures were performed under 4°C.

Cell disruption was performed by ultrasonication [38], high-pressure homogenization [46], grinding in mortar [66], or disintegration with glass beads [63] to prepare cell-free extracts. The cell lysate was centrifuged to remove cell debris. In *F. solani* O1 nitrilase, up to 90% of the whole-cell activity was recovered in cell-free extract using an extraction buffer supplemented with 0.8 M ammonium sulfate. A large ratio of contaminating cellular protein was removed during the extraction [67]. Furthermore, the purification process was performed using ammonium sulfate fractionation and various types of column chromatography techniques. Table 3 lists some reported information on the purification procedures of nitrilase.

**Table 3:** Reported purification procedures of nitrilase

**Organisms**	**Purification process**^**a**^**(In order)**	**Purification fold**	**Specific activity (U·mg**^**-1**^**)**	**Yield (%)**	**Reference**
*Pseudomonas* sp.	DEAE-cellulose; Ammonium sulfate fractionation; Starch electrophoresis	400	12	14	[28]
*Nocardia* sp. NCIB 11215	DEAE-cellulose; Ammonium sulfate fractionation; Sephadex G-200	15.82	1.74	9.1	[123]
*Pseudomonas* sp. 13	Ethanol fractionation; Ammonium sulfate fractionation; DEAE-cellulose; 1^st^ crystallization; 2^nd^ crystallization	656	3280	21	[31]
*F. oxysporum* f sp. *melonis*	DEAE-Sephacel; TSK-phenyl 5 PW	39.72	143	50	[64]
*R. rhodochrous* K22	DEAE-Sephacel; Ammonium sulfate fractionation; Phenyl-Sepharose CL-4B; Cellulofine GCL-2000 superfine	8.3	0.737	9.08	[40]
*Acinetobacter* sp. AK 226	1^st^ DEAE-cellulose; 1^st^ Hydroxyapatite; 2^nd^ DEAE-cellulose; 2^nd^ Hydroxyapatite; Sephacryl S-400	4.41	0.156	20.1	[37]
*A. faecalis* ATCC 8750	Ammonium sulfate fractionation; Phenyl-Sepharose CL-4B; DEAE-cellulose	29.0	3.10	17.9	[38]
*C. testosteroni* sp.	Q-Sepharose; Superdex 200; Hydroxyapatite	60	68	79	[35]
*R. rhodochrous* NCIMB 11216	Ammonium sulfate precipitation; Anion-exchange FPLC; Sepharose Q; Suparose 12 HR 10/30	62	10.6	9.5	[43]
*R. rhodochrous* PA-34	Ammonium sulfate fractionation; 1^st^ Sephacryl S-300 HR; 2^nd^ Sephacryl S-300 HR; DEAE Toyopearl 650S	14.10	3.52	34.8	[39]
*P. fluorescens* DSM 7155	Phenyl-Sepharose FF; Mono Q; Superose 12	259	90	10	[44]
*A. facilis* 72W	Ammonium sulfate precipitation; Sephadex G-25; Q-Sepharose	10.5	21	65	[48]
*P. putida* MTCC 5110	Ammonium sulfate fractionation; Superdex 200; Q-sepharose; Phenyl sepharose	35.01	3.26	10.15	[46]
*A. niger* K10	Ammonium sulfate precipitation; Sephacryl S-200; Q-Sepharose	18.7	91.6	24.3	[66]
*P. abyssi* GE5	Heat treatment; Q-Sepharose	23.85	141.9	88	[47]
*F. solani* O1	Phenyl sepharose; Sephacryl S-200; Q-Sepharose	9.9	156	25.9	[67]
*F. solani* IMI 196840	Phenyl sepharose; Sephacryl S-200; Q-Sepharose	20.3	144.0	26.9	[69]

^a^ Purification process only listed the adopted column chromatography techniques after cell-free extracts preparation.

### Immobilization of nitrilase

Immobilization of whole cells and purified enzymes can make the biocatalytic process more economical. To our knowledge, most industrial processes for nitrile biocatalysis were performed using immobilized biocatalysts. However, little information is reported on the biotransformation of nitriles by free cells in commercial scale.

The availability of immobilized biocatalysts being recycled can greatly reduce the operational cost in the production process. Compared with the free catalysts, separating the immobilized biocatalysts from the bioconversion reaction mixture is easier, making the repeated batch operations possible and simple. The activity of immobilized lipase in sol–gel was enhanced by up to 100-fold compared with the free ones [124]. Moreover, immobilization improved the operational stability, and immobilized cells were found to hydrolyze a wider range of nitriles than free cells [125]. Hydrolysis may have contributed to the protective role of immobilized cells against the toxic effect of nitrile substrates. A greater reaction rate than that of free cells was observed for immobilized *A. faecalis* MTCC 126 nitrilase at 65°C [126]. This nitrilase of free cells was extremely unstable, and thus it was conjectured that its thermostability increased significantly after immobilization. Meanwhile, immobilized *Candida guilliermondii* CCT 7207 cells degraded some nitriles that cannot be utilized by the corresponding free cells [127].

A wide variety of immobilization methods and materials were reported in numerous studies [126-128]. The immobilization methods mainly include entrapment, cross-linking, adsorption, and covalent bonding. The immobilization materials include polyvinyl alcohol, DEAE cellulose, alginate (Sr^2+^, Ba^2+^, Na^+^, Al^3+^, Ca^2+^, and so on), alumina, carrageenan gels, and polyacrylamide. Notwithstanding the immobilization procedure can lead to the partial loss of enzyme activity, numerous studies on nitrilase-mediated biocatalysis by immobilized catalysts were carried out. The *P. putida* nitrilase activity of cross-linked enzyme aggregate preparation remained stable for 75 h at 4°C, whereas the free enzyme lost 90% nitrilase activity at the same temperature for 75 h [129]. Liu et al. used polyvinyl alcohol and alginate (Na^+^) copolymer to entrap the recombinant cells harboring *A. facilis* nitrilase [128]. The entrapped cells were firstly utilized in iminodiacetic acid preparation from iminodiacetonitrile and showed moderately good operation stability. The immobilized biocatalyst can be reused for 10 batches, whereas the free cells completely lost the activity after 9 batches. Immobilized *Streptomyces* nitrilase in a cheap matrix (agar-agar) was applied in the biotransformation of acrylonitrile at 50°C in a batch operation mode [56]. The immobilized cells can be reused for 25 cycles, with only an approximately 20% enzyme activity loss. Immobilization using alginate resulted in efficient recycling of the *A. faecalis* nitrilase, retaining almost 100% activity up to 30 consecutive cycles compared with only nine cycles for free cells [126]. Also, the recycle efficiency can be further increased to 40 batch reactions through cross-linking of alginate beads using glutaraldehyde and polyethylene imine.

### Molecular cloning and modification of nitrilase gene

#### Gene cloning and heterologous expression

Enzyme reengineering becomes a common process with the development of modern molecular biology and the requirement of more efficient biocatalytic reaction. Natural biocatalysts often cannot meet the expectations of scientists and engineers with respect to some enzymatic properties. Therefore, constructing a recombinant enzyme combining with some gene manipulation, such as direct evolution, would provide the possibility to further meet the synthetic application requirement with high efficiency. Several nitrilase genes are presently cloned from various organisms and introduced into appropriate host strains.

As early as 1987, Stalker et al. successfully cloned the gene locus (*bxn*) that encode the bromoxynil-specific nitrilase in *K. ozaenae*[108], and this *bxn* gene was ligated with pUC18 vector and overexpressed in *E. coli* 71*–*18. Shortly thereafter, the first plant gene from *A. thaliana* coding nitrilase was identified, and an *A. thaliana* nitrilase cDNA was isolated, characterized, sequenced, and functionally expressed [130]. On the other hand, Pekarsky et al. cloned and characterized tumor suppressor genes from *D. melanogaster* and *C. elegans*[131], and their fusion protein showed homology to bacterial and plant nitrilases.

Kobayashi and his co-workers cloned and sequenced the gene for nitrilase from *A. faecalis* JM3 and expressed the active enzyme in *E. coli*[132]. A 35% increase in the specific activity, together with an obvious decrease of the *K*m value for thiophene-2-acetonitrile, was observed. In addition, the study strongly proved that Cys-163, one of the five cysteine residues in the nitrilase, was crucial for the function of the active site. The same hypothesis was also supported by the experimental results for recombinant nitrilases from *C. testosteroni* sp. [35] and *R. rhodochrous* K22 [133]. A regioselective aliphatic nitrilase from *A. facilis* 72W was cloned and overexpressed under the control of strong T7 promoter in the pET-3c vector [48]. The engineered nitrilase displayed a 15-fold higher enzyme activity than the native 72W strain; however, the increase of specific activity was approximately three fold. The protein engineering combined with fermentation optimization for this recombinant nitrilase of *A. facilis* was employed to improve catalytic efficiency for glycolic acid production, and the specific enzyme activity was increased up to 125-fold [134]. Zhu et al. cloned and overexpressed a novel nitrilase gene blr3397 from *B. japonicum* USDA 110 in *E. coli*[135], which was proven extremely specific towards hydrocinnamonitrile (about 9.99 U·mg^-1^ protein). Mueller et al. cloned and expressed a thermoactive nitrilase from *P. abyssi* in *E. coli*[47]. The recombinant enzyme was proven highly thermostable, with a half-life of 6 h at 90°C.

The heterologous expression of genes that encode nitrilases from filamentous fungi has scarcely been investigated. Until recently, a fungal nitrilase gene of *A. niger* K10 was amplified from the cDNA library and ligated into expression vectors pET-30a(+) (Novagen) and pRSET B (Invitrogen). A two fold increase was observed in the specific activity of recombinant nitrilase (Nit-ANigRec) expressed in *E. coli* BL21-Gold(DE3) (pOK101/pTf16) [68]. Moreover, genome mining was performed to search for fungal nitrilases [136], and synthetic genes of putative nitrilase from *Gibberella*, *Neurospora*, and *Aspergillus* were expressed in *E. coli*.

#### Directed evolution

The commercial availability of biocatalysts is a limiting factor for their practical application. Heterologous expression of nitrilase gene in host cells becomes a routine manner. In recent years, directed evolution was rapidly emerging as a novel and powerful strategy for the improvement of the catalytic properties of enzymes, such as specific activity, substrate tolerance, and thermostability. This technique mainly include two steps: establishing random mutation libraries through error-prone polymerase chain reaction (EP- PCR) as well as DNA recombination and screening of libraries using various chemical methods. Schreiner et al. used EP-PCR with recombination of beneficial mutations to improve both specific activity of nitrilase from *A. faecalis* JM3 and its fitness at low pH values [137]. The nitrilase activity of individual clones from mutation libraries was determined in a 96-well microtiter plate through the colorimetric reaction of the Berthelot method. A variant with eight fold improvement of the specific activity for 2-phenylpropionitrile at pH 7.5 was ultimately obtained. The sequence analysis showed that this variant carried seven mutations. Furthermore, another variant (pHNIT45) was proven to harbor high activity at pH values as low as pH 4.5. pHNIT45 can fully convert 10 mM (*R*)-2-Cl-mandelonitrile into (*R*)-2-Cl-mandelic acid stoichiometrically, with enantiomeric excess (*ee*) > 99% within 10 min.

#### Site-directed mutagenesis

Site-directed mutagenesis for nitrilase modification at the gene level attracted increasing interest in some recent studies. Point mutations, even a single amino acid exchange, may create a significant impact on specific activity, substrate specificity, selectivity, amide formation capacity, and amide:acid ratio. Tyr-142 in the nitrilase gene from *R. rhodochrous* ATCC 33278 is an important amino acid residue for substrate specificity [138]. The mutants that contain non-polar aliphatic amino acid at this position were specific only for aromatic nitriles but not for aliphatic ones. The substrates hydrolysis probably needed a conjugated π-electron system in nitriles or Tyr-142 residue. Ala-165 and Cys-163 residues in the *P. fluorescens* EBC191 nitrilase, which was in close proximity to the cysteine residue in the catalytic center, were identified and proven responsible for the enantioselectivity and amide formation [139]. Especially, the mutation C163Q resulted in an obvious increase in amide formation, but reduced enzyme activities towards (*R*)-mandelonitrile. Moreover, deletion mutants with 47–67 C-terminal amino acids missing displayed reduced activities, increased amide formation and changes of enantiospecificity [97]. Thus, the combined mutants of these mutations were constructed and the results demonstrated that the combination led to a 1.5-fold increase in mandeloamide formation and exhibited the highest NHase activity among all the variants of *P. fluorescens* nitrilase [140]. Petříčková et al. recently identified the W168A mutant of *Neurospora crassa* nitrilase and observed significant increase in amide generation and decrease of *ee* value for the products [141].

### Biotechnological potential of nitrilase

Biocatalysis has long been seen by biochemists and microbiologists as an area with greater potential for chemical synthesis over conventional chemical processes [9]. A considerable amount of studies on nitrile biotransformation were carried out over the last few decades. Among them, nitrilase was proven a valuable candidate, and microbial cells were considered a potential producer for their scientific and industrial aspects. Moreover, microbial cells harboring nitrilase as a factory can be used to hydrolyze nitrile compounds into carboxylic acids and their derivatives. In this area, one of the most inspiring stories of success is the biosynthesis of acrylamide using NHase on a commercial scale, with an annual production exceeding 100,000 tons. Some nitrilases have also been successfully applied to practical production in food industries, chemical manufacturing, pharmaceutical processes, wastewater treatment and textile industries.

### Application in carboxylic acid production

#### Nicotinic acid

**Figure 4:**
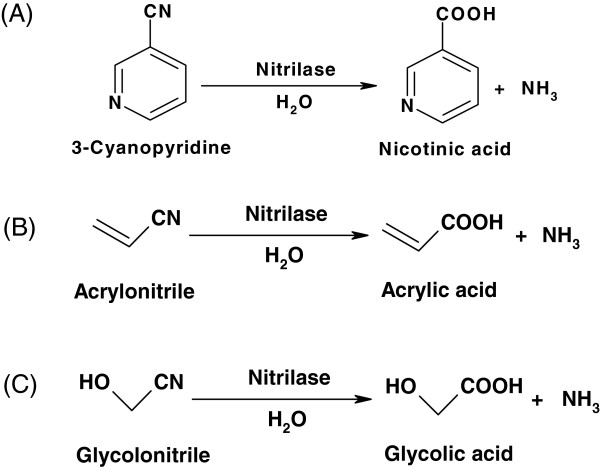
Biosynthesis of nicotinic acid (A), acrylic acid (B), and glycolic acid (C) with nitrilase, respectively.

Nicotinic acid, also known as niacin or vitamin B3, is often used in the production of feedstuff additives and pharmaceutical intermediates. The conventional method for preparing nicotinic acid was established over 100 years ago through oxidizing nicotine with potassium dichromate [142]. To date, the microbial transformation of 3-cyanopyridine was attempted to synthesize nicotinic acid (Figure 4A). Mathew et al. reported the use of free cells of *R. rhodochrous* Jl to directly catalyze of 3-cyanopyridine to nicotinic acid, and the highest yield achieved was 172 g·L^-1^ nicotinic acid with 100% conversion rate of 3-cyanopyridine [33]. Alginate-immobilized *B. pallidus* Dac521 cells were used to continuously hydrolyze 3-cyanopyridine to nicotinic acid, and the conversion efficiency of about 104 mg (substrate)·g (cells)^-1^·h^-1^ and 208 mg (substrate)·g (cells)^-1^·h^-1^ was observed at 50°C and 60°C, respectively, in the initial 10 h [121]. Fed-batch reaction was employed by Sharma et al. to prepare nicotinic acid through the *N. globerula* NHB-2 nitrilase, and the productivity of 3.21 mg (nicotinic acid)·mg (cells)^-1^·h^-1^ was recorded in their study [143]. Prasad and co-workers revealed that the propionitrile-induced nitrilase of *Rhodococcus* sp. NDB 1165 can efficiently synthesize nicotinic acid at the rate of 8.95 mg (product)·mg (cells)^-1^·h^-1^, and the highest yield achieved reached 196.8 g·L^-1^ nicotinic acid [49]. Lonza, the world’s largest manufacturer of nicotinates, made an investment in China to produce nicotinic acid in a commercial scale [23,144].

#### (*R*)-(−)-mandelic acid

**Figure 5:**
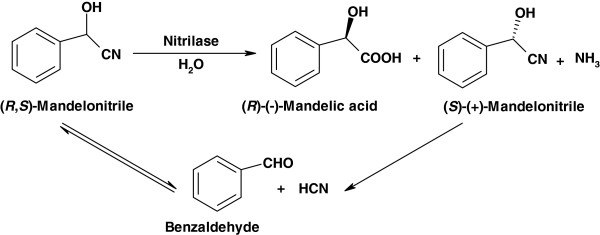
**Biosynthesis of (*R*)-(−)-mandelic acid from (*R,S*)-mandelonitrile with nitrilase.**

(*R*)-(−)-mandelic acid is an important chiral building block, broadly used as optical resolving agents and intermediates for the production of various pharmaceutical and agricultural products [145,146]. (*R*)-(−)-mandelic acid production can be performed using physicochemical methods and biocatalytic routes: the latter usually use (*RS*)-mandelonitrile as substrate. In recent few years, the enantioselective biocatalytic routes for the production of (*R*)-(−)-mandelic acid with nitrilase from (*RS*)-mandelonitrile were widely investigated by several groups, such as the Xu’s and the Zheng’s in China. This hydrolysis reaction by nitrilase generally proceeded to generate (*S*)-(+)-mandelonitrile and (*R*)-(−)-mandelic acid, whereas the residual (*S*)-(+)-mandelonitrile in the reaction mixture was spontaneously racemized due to the chemical equilibrium and used as substrate over again, as shown in Figure 5[60]. As illustrated by the Xu’s group, the recombinant *Alcaligenes* sp. nitrilase overexpressed in *E. coli* displayed an excellent specific activity of 7000 U·L^-1^ toward (*RS*)-mandelonitrile, and 520 mM (*R*)-(−)-mandelic acid was produced after 17.5 h of conversion with 600 mM (*RS*)-mandelonitrile in a fed-batch reaction, in which the volumetric productivity and catalyst productivity reached up to 4.5 g (product)·L^-1^·h^-1^ and 0.79 g (product)·g (cells)^-1^, respectively, with *ee* value of 99% [122]. These authors recently employed the toluene-water biphasic system to relieve substrate inhibition [147]. Finally, the average productivity of 352.6 g (product)·L^-1^·d^-1^ was achieved, and 110.7 g (*R*)-(−)-mandelic acid in 98.0% *ee* along with the catalyst productivity of 13.8 g (product)·g (cells)^-1^ can be obtained when immobilized cells were repeatedly used for five batches in a 2-L stirred reactor. Moreover, the mutant of *A. faecalis* obtained through ultraviolet irradiation and N^+^ ion beam implantation technique by Zheng’s group showed considerable potential in (*R*)-(−)-mandelic acid production, and its highest production rate reached 9.3 mM (product)·g (cells)^-1^·h^-1^ with 93% yield and *ee* value of more than 99% [60]. Meanwhile, *Saccharomyces cerevisiae* FD11b was used by Xiao et al. to asymmetrically reduce the phenylglyoxylic acid to (*R*)-(−)-mandelic acid where the maximal production rate reached 0.353 mM (product)·g (cells)^-1^·h^-1^, and the highest product concentration achieved about 120 mM with an *ee* value of 97.1% [148]. BASF in Germany and Mitsubishi Rayon in Japan are currently producing (*R*)-(−)-mandelic acid from (*RS*)-mandelonitrile with at least several tons per year [9,24,25,146].

#### Acrylic acid

Acrylic acid, also known as 2-propenoic acid, is a principal commodity chemical with an estimated annual output of 4.2 million metric tons [149]. Acrylic acid and its derivatives are widely used in surface coatings, textiles, adhesives, paper treatment, polymeric flocculants, dispersants, and so on [150]. Traditionally, commercial acrylic acid was produced from petrochemical industry using the two-step gas-phase oxidation of propylene, in which several of the by-products, such as acrolein and large quantities of inorganic wastes, were unexpectedly generated [57]. Furthermore, acrylic acid may be prepared through fermentative production with sugars as a raw material [149]. The enzymatic hydrolysis of acrylonitrile using nitrilase is currently considered an attractive alternative for the synthesis of acrylic acid (Figure 4B). Very recently, a novel acrylonitrile-degrading *A. nitroguajacolicus* was isolated and characterized [57], and its enzymatic properties confirmed its application potential in acrylic acid production (specific activity of 202.24 U·g (cells)^-1^, half-life of 129.8 h at 40°C). Zabaznaya et al. adopted a bacterial strain of *Alcaligenes* sp. for transforming acrylonitrile, and finally 115 g·L^-1^ acrylic acid was obtained over a 9 h reaction [151]. Moreover, the *R. rhodochrous* J1 nitrilase induced by ε-caprolactam was extremely specific toward acrylonitrile [152]. The highest accumulation of 390 g·L^-1^ acrylic acid was attained through transformation reaction mediated by this nitrilase. A mutant strain of *R. rhodochrous* tg1-A6, which was obtained after treatment through UV light combined with lithium chlorinate, was used to synthesize acrylic acid [153]. The results demonstrated that a final concentration of 414.5 g·L^-1^ acrylic acid was accumulated during a 10 h continuous catalytic reaction.

#### Glycolic acid

In recent years, α-hydroxycarboxylic acids have become significant because of their important medical and pharmaceutical applications. These acids are broadly used in household cleaners, industrial and electronic elements, automotive oil additives, oil and water well flow enhancers, pH controlling, cosmetics, and many others [154]. Glycolic acid, the simplest kind of α-hydroxycarboxylic acid, has been a subject of several studies due to its large demand and wide application. Majority of the commercially available glycolic acid are currently produced using high-pressure and high-temperature reaction of formaldehyde and carbon monoxide under acid catalysis [155]. Recently, several studies focused on the enzymatic transformation of glycolonitrile to glycolic acid (Figure 4C) [105,107,134,155]. The GA/PEI cross-linked carrageenan immobilized cells of *Alcaligenes* sp. were applied in glycolonitrile conversion [105]. A volumetric productivity of 18.0 g (product)·L^-1^·h^-1^ and total productivity of 1,042.2 g (product)·g (cells)^-1^ were achieved after 29 cycles. The nitrilase from *A. facilis* 72W was overexpressed and protein engineered to improve the catalytic efficiency for glycolonitrile [134]. An F168V mutant demonstrated the best performance, with a productivity of 1,010 g (product)·g (cells)^-1^ after 55 recycle reactions.

### Bioremediation and biodegradation potential

Nitriles can be degraded in the environmental contaminants by enzymatic reaction. This degradation is of great significance because most nitrile compounds are highly toxic, carcinogenic, and mutagenic in nature, which are considered harmful to human beings, animals, and plants [98]. These compounds typically included acetonitrile, acrylonitrile, succinonitrile, benzonitrile, dichlobenil, bromoxynil, and ioxynil, which are often used in industry and agriculture for pharmaceuticals, plastics, synthetic rubbers, herbicides, and pesticides [13,98]. The bioremediation potential of nitrilase has been illustrated in detail to degrade the compounds into harmless intermediates or, ultimately, carbon dioxide, as well as H_2_O. Several microorganisms are involved in the degradation of nitriles in literature [13,16,17,156]. Zhou et al. reported that sodium alginate immobilized the microbial cells, which were isolated from acrylic fiber production wastewater and can degrade more than 80% of succinonitrile at the initial concentration of even 5,000 mg·L^-1^ after 24 h [156]. *K. pneumoniae* subsp. *ozaenae* was obtained by enrichment strategies with the herbicide bromoxynil as a nitrogen source, and was found capable of rapid conversion of 0.05% bromoxynil to 3,5-dibromo-4-hydroxybenzoic acid [157]. The nitrilase gene was cloned and expressed in *E. coli*, and the recombinant nitrilase was highly specific for bromoxynil with *V*_max_ of 15 μM (NH_3_)·mg (protein)^-1^·min^-1^[41]. The soil actinobacteria *R. rhodochrous* PA-34, *Rhodococcus* sp. NDB 1165, and *N. globerula* NHB-2 exhibited excellent specific activities towards benzonitrile [16]. These bacteria can hydrolyze chloroxynil and bromoxynil completely, whereas 60% of ioxynil with 0.5 mM substrate concentration is hydrolyzed into corresponding acids after 20 h incubation. Other strains are also reported to have significant nitrilase activity for nitrile bioremediation, such as the genus of *Alcaligenes*, *Bacillus*, *Stemphylium*, *Fusarium*, *Pseudomonas*, and *Arthrobacter*[17,158].

### Surface modification of polymers

Approximately 2.73 million tons of polyacrylonitrile (PAN) are produced worldwide by polymerization of acrylonitrile per year [159]. Most of these PAN were used as fibers in textile processing. Conventional chemical methods have difficulty to improve the quality and the processing properties of fibers. The treatment using strong acids or alkali, high reaction temperatures, aggressive chemicals, and high concentrations of dimethyl sulfoxide would lead to irreversible yellowing and unwanted changes in the macroscopic behavior of the fabrics [160]. Therefore, surface modification through selective enzymatic hydrolysis is a more promising alternative to specifically modify the PAN surface to make fibers more hydrophilic and enhance the dye uptake. NHases are regularly adopted to selectively modify the PAN surface groups, such as those from *B. imperiale* CBS 49874 and *Corynebacterium nitrilophilus* ATCC 21419 [161]. Information on the surface modification mediated by nitrilase is rare. Matamá et al. described the first case of direct enzymatic modification of -CN groups into -COOH on fibres using a commercial nitrilase [162]. Their experimental results showed that the staining level of samples treated with nitrilase increased by 156% compared with the control, and 199% of the increase can be observed by the addition of 1 M sorbitol and 4% *N,N*-dimethylacetamide. Almost simultaneously, *Micrococcus luteus* BST20 was isolated by Fischer-Colbrie et al. with PAN polymers as sole carbon source [159]. The staining depth of the PAN fabrics was substantially improved when treated by this catalyst. The nitrilase from *E. coli* BL21 (DE3)/pET-Nit can also effectively modify the surface of PAN fibers and membrane [163]. The hydrophilicity and fabric-dyeing efficiency of these fibers were obviously increased and yet the strength of treated fiber decreased by only 1.17%.

### Major challenges faced by researchers

Nitrilase-mediated biocatalysis has undergone fast development, and it is expected that some limitations would be overcome in the future. The biocatalytic process develops biocatalysts with appropriate activities and stability as a “top priority”. Therefore, the present and future research efforts would be focused on improving enzyme properties, such as substrate spectrum, stability, specificity, and function in non-classical environments [9].

The specific activity of most nitrilases is lower than that of commercial application. An extremely small proportion of native strains and protein sequences is presently available to the scientific community [164]. The screening and discovery of novel nitrilase-producing strains or new nitrile converting enzymes with greater potential is still currently conducted.

Second, the substrate spectrum of nitrilase needed to be widened. In the long term, narrow substrate spectrum is a limiting factor in the development of general purpose catalysts [6]. Nitrilases that can degrade a wide range of nitrile substrates to various carboxylic acids, would become a research interest of chemists and biotechnologists.

Third, operational stability should be improved from the industrial application perspective. The operational stability of nitrilase can be increased through the isolation of stable wild type strains using certain screening methods and then reengineering the target strain toward better stability through gene modification [165].

Finally, biocatalysis reaction in organic solvents would become a hot topic in this area. Nitrile compounds are poorly soluble in water or buffer solutions, thus, organic solvents can increase the solubility of substrates, thereby prompting catalytic efficiency. Nevertheless most organic solvents may affect the protein structure or destroy the enzyme activity. Therefore, enzymatic catalysis in organic solvents requires further investigations.

## Conclusions and future prospects

Nitrilase-producing organisms are useful cell factories for the production of commercially important carboxylic acids. The waste treatment and textile industry also have drawn benefits from nitrilase due to its bioremediation and surface modification functions. The microbiological, enzymological, and molecular biological aspects of nitrilase were investigated by a large number of scholars and have made its industrial application an attractive alternative. Overcoming the disadvantages of nitrilase would further widen its application in organic synthesis. Meanwhile, new techniques have been introduced and developed in the recent years; thus researchers have gained greater opportunities in promoting nitrilase as a more promising biocatalyst. Molecular biology technique, genetic engineering, immobilization, enzyme engineering, enzyme crystallography, and process engineering would receive increasing attention for the improvement of the catalytic properties of nitrilase. The research for extreme microorganisms including those of thermophile, alkali/acid resistance, and substrate tolerance would extend the application scope of nitrilase [21,166]. High throughput screening is widely applied in the selection of improved biocatalysts. Some novel strategies, including rational protein design, as well as the combination of the rational design and directed evolution [10] are worthy of reconsideration for a versatile and powerful biocatalyst with wide substrate spectrum, efficient catalytic activity, and excellent operational stability. Inevitably, broader application of nitrilase is expected in the near future.

## Abbreviations

NHase: Nitrile hydratase; Bromoxynil: 3,5-dibromo-4-hydroxybenzonitrile; Ioxynil: 3,5-diiodo-4-hydroxybenzonitrile; AtNIT: *Arabidopsis thaliana* nitrilase; PAN: Polyacrylonitrile; IAN: Indole-3-acetonitrile; IAA: Indole-3-acetic acid; NMR: Nuclear magnetic resonance; CD: Circular dichroism; EDTA: Ethylene diamine tetra acetic acid; EP- PCR: Error-prone polymerase chain reaction.

## Competing interests

The authors declare that they have no competing interests.

## Authors’ contributions

All authors defined the topic of the review and wrote, read and approved the manuscript.
